# Equivalent 2-year stabilization of uncemented tibial component migration despite higher early migration compared with cemented fixation: an RSA study on 360 total knee arthroplasties

**DOI:** 10.1080/17453674.2018.1562633

**Published:** 2019-01-23

**Authors:** Elise K Laende, Janie L Astephen Wilson, Joanna Mills Flemming, Edward R Valstar, C Glen Richardson, Michael J Dunbar

**Affiliations:** a School of Biomedical Engineering, Dalhousie University, Halifax, Nova Scotia, Canada;;; b Division of Orthopaedics, Department of Surgery, Dalhousie University and QEII Health Sciences Centre, Nova Scotia Health Authority, Halifax, Nova Scotia, Canada;;; c Department of Surgery, Dalhousie University, Halifax, Nova Scotia, Canada;;; d Department of Mathematics and Statistics, Dalhousie University, Halifax, Nova Scotia, Canada;;; e Department of Orthopedics, Leiden University Medical Center, Leiden, The Netherlands

## Abstract

Background and purpose — Thresholds of implant migration for predicting long-term successful fixation of tibial components in total knee arthroplasty have not separated cemented and uncemented fixation. We compared implant migration of cemented and uncemented components at 1 year and as the change in migration from 1 to 2 years.

Patients and methods — Implant migration of 360 tibial components measured using radiostereometric analysis was compared at 1 year and as the change in migration from 1 to 2 years in 222 cemented components (3 implant designs) and 138 uncemented components (5 implant designs).

Results — 1-year maximum total point motion was lower for the cemented tibial components compared with the uncemented components (median = 0.31 mm [0.03–2.98] versus 0.63 mm [0.11–5.19] respectively, p < 0.001, mixed model). The change in migration from 1 to 2 years, however, was equivalent for cemented and uncemented components (mean [SD] 0.06 mm [0.19] and 0.07 mm [0.27] mm respectively, p = 0.6, mixed model).

Interpretation — These findings suggest that current thresholds of acceptable migration at 1 year may be better optimized by considering cemented and uncemented tibial components separately as higher early migration of uncemented components was not associated with decreased stability from 1 to 2 years.

Cemented fixation in total knee arthroplasty (TKA) is currently the most common method of fixation, but there is increasing interest in uncemented TKA in an effort to provide longer lasting constructs to the young, active patient through osseointegration of the tibial component (Drexler et al. 2012, Brown et al. 2013, Cherian et al. 2014, Mont et al. 2014). A concern with uncemented TKA is that failure to achieve initial fixation may lead to revisions due to aseptic loosening. Early patterns of implant migration measured with radiostereometric analysis (RSA) have been shown to predict long-term implant outcomes. In particular, 2 studies have demonstrated the predictive value of migration 1-year post-operation (Pijls et al. 2012b), and the change in migration between 1 and 2 years postoperatively (Ryd et al. 1995) in determining long-term survivorship. Notably, both of these studies pooled cemented and uncemented tibial components in their analyses. In contrast, a Cochrane Review (Nakama et al. 2012) concluded that although cemented tibial components had lower initial migration, uncemented fixation provided a lower risk of future aseptic loosening, as measured indirectly as a change in migration between 1 and 2 years, despite higher early migration. While cemented fixation depends on an immediate mechanical interlock provided by cured bone cement, uncemented fixation requires bone in-growth into the implant surface, which occurs in the early postoperative period (Freeman and Tennant 1992, Dalury 2016). Because of these fundamental differences in the mechanisms of early fixation for cemented and uncemented components, it is unclear if it is appropriate to evaluate cemented and uncemented tibial components under the same thresholds of early migration for prediction of successful fixation.

In this study we compared the magnitudes of implant migration of cemented and uncemented tibial components at 1 year postoperatively and between 1 and 2 years postoperatively. We hypothesized that the uncemented components would have higher migration levels at 1 year postoperatively, but similar migration magnitudes between 1 and 2 years postoperatively, indicating good long-term performance. The secondary objective of this analysis was to examine the effect of implant design on tibial component migration.

## Methods

This study included RSA data on subjects who received a primary TKA between 2002 and 2015 at 2 institutions (Halifax Infirmary, Halifax, Nova Scotia and St. John of God Hospital Subiaco, Perth, Australia). The source of data for this study is the Halifax RSA Database, which was created with the aim of collecting RSA outcome data on a wide range of arthroplasty implants. Subjects were included in the database if they were part of implant-specific RSA protocols (completed or ongoing) or were enrolled in an implant-generic RSA protocol for any subject undergoing primary or revision knee or hip arthroplasty (Figure 1 and Tables 1 and 2, see Supplementary data).

**Figure 2.**
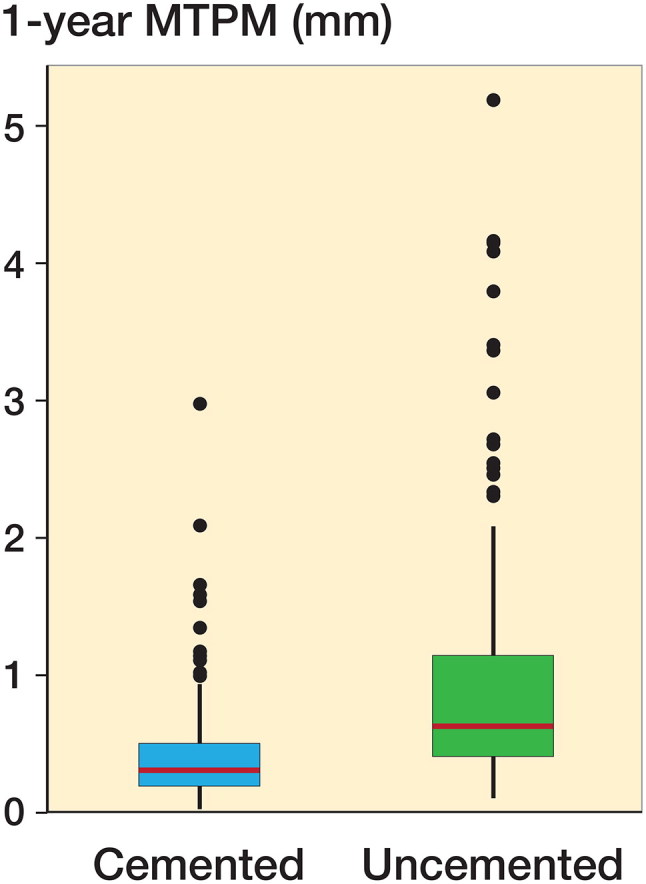
1-year MTPM migration by fixation (cemented, n = 222; uncemented, n = 138). Boxes enclose 25th–75th percentiles with internal horizontal line at the median, whiskers extend a further 1.5 times the inter-quartile range and points beyond this range are plotted individually.

**Table 6.** Subject demographics by fixation (cemented and uncemented) and by implant design

Implant	Fixation	Insert^a^		n	Mean age (SD)	Mean BMI (SD)	% female
All implants				360	64 (7.8)	32 (6.1)	61
All cemented implants				222	64 (8.3)	33 (6.5)	68
Advance^b^	MP, PS			59	64 (7.8)	32 (5.6)	69
NexGen^c^	PS			30	66 (8.7)	32 (5.7)	60
Triathlon^d^	PS, CR, CS			133	64 (8.4)	34 (6.9)	70
All uncemented implants				138	65 (7)	31 (5.2)	49
Advance Biofoam^b^	porous coated, no screws	MP		22	69 (5.2)	30 (3.8)	55
Advance Biofoam^b^	porous coated + screws	MP		20	69 (5.2)	31 (4.6)	35
TM Monoblock^c^	trabecular metal	PS, CR		48	64 (7.7)	32 (5.4)	60
TM Modular^c^	trabecular metal	PS		16	62 (8.7)	35 (4.8)	63
Triathlon PA-Coated^d^	porous coated + periapatite	PS, CR, CS		32	65 (8.4)	29 (5.0)	31

a MP = medial pivot (posterior cruciate ligament resected), PS = posterior stabilized (posterior cruciate ligament resected),

CR = cruciate retaining, and CS = cruciate stabilized.

b Wright Medical Technology, Inc., Arlington, TN

c Zimmer, Warsaw, IN

d Stryker, Mahwah, NJ

**Table 7.** Tibial component 1-year MTPM migration and change in MTPM migration from 1 to 2 years by fixation and implant groups

Implant	n	1-year migration Mean (SD)	(MTPM, mm) Median (range)	Change in MTPM from Mean (SD)	1 to 2 years (mm) Median (range)
All cemented implants	222	0.41 (0.36)	0.31 (0.03–2.98)	0.06 (0.19)	0.04 (–0.38 to 1.76)
Advance	59	0.40 (0.24)	0.30 (0.16–1.13)	0.04 (0.14)	0.06 (–0.31 to 0.36)
NexGen	30	0.49 (0.34)	0.38 (0.14–1.66)	0.13 (0.38)	–0.01 (–0.16 to 1.76)
Triathlon	133	0.40 (0.41)	0.28 (0.03–2.98)	0.05 (0.14)	0.03 (–0.38 to 0.53)
All uncemented implants	138	0.98 (0.94)	0.63 (0.11–5.19)	0.07 (0.27)	0.04 (–0.76 to 1.30)
Biofoam	22	1.08 (1.05)	0.68 (0.18–4.09)	0.05 (0.32)	0.01 (–0.62 to 1.25)
Biofoam + screws	20	0.82 (0.75)	0.66 (0.29–3.80)	0.04 (0.31)	0.04 (–0.55 to 0.94)
TM Monoblock	48	0.89 (0.77)	0.55 (0.14–3.06)	0.05 (0.21)	0.04 (–0.76 to 0.78)
TM Modular	16	1.52 (1.24)	1.20 (0.31–5.19)	0.32 (0.35)	0.19 (–0.10 to 1.30)
Triathlon PA	32	0.85 (1.00)	0.50 (0.11–4.17)	–0.01 (0.19)	0.00 (–0.72 to 0.43)

All subjects had tantalum RSA markers inserted into the proximal tibia and into the non-articulating periphery of the polyethylene component at the time of surgery.

Subjects received postoperative care that included antibiotics, anticoagulation medication, and physiotherapy in hospital and after discharge. All subjects were mobilized to immediate full weight-bearing postoperatively.

Subjects were followed for 2 years and had RSA exams immediately postoperatively (reference exam) and at a minimum of 1 and 2 years postoperatively. Details of the RSA equipment are included in the Supplementary data (Table 3). Inclusion criteria for this analysis were a primary diagnosis of osteoarthritis, no previous knee replacement, and RSA migration data at both 1 and 2 years postoperatively. Exclusion criteria included severe joint deformity requiring revision components in primary cases, revision of the tibial component, and technical problems with the RSA analysis (insufficient markers visible, condition number > 150, or mean error of rigid body fitting > 0.35 mm) (Valstar et al. 2005).

The primary outcome measure was RSA-defined implant migration calculated as maximum total point motion (MTPM), the vector length of the point on the implant that moved the most (Ryd et al. 1995). All analyses used fictive markers at standardized locations for MTPM calculations (Nilsson et al. 1991). Rigid body motions were calculated using marker-based methods (Selvik 1989) to eliminate any differences due to model fitting that may occur with model-based RSA. Migrations at 1 and 2 years were calculated relative to the immediate postoperative reference exam.

### Statistics

Mixed models were fitted to determine whether fixation (cemented or uncemented) had a significant effect on (i) migration at 1 year (relative to the immediate postoperative reference exam) and (ii) the change in migration between 1 and 2 years postoperatively. The models included patients as random effects (to account for bilateral cases) along with fixed effects for sex, age, and BMI using the lme4 package (Bates et al. 2015) in R (R Core Team 2015). For 1-year migrations, log(MTPM) was taken as for the outcome variable (Astephen et al. 2010, Pijls et al. 2012a). For the change in migration from 1 to 2 years, the proportion of subjects for which it exceeded the 0.2 mm threshold (indicative of continuous migration) was also calculated (Ryd et al. 1995). Similar mixed models were fitted to the cemented and uncemented groups separately in order to investigate the influence of implant design. Significance was set at p < 0.05. Confidence intervals for statistical analyses are included in the Supplementary data (Table 5).

### Ethics, funding, and potential conflict of interest

Ethics approval was obtained (local REB approval number 1020265) and subjects provided written consent. Funding was provided by the Atlantic Canada Opportunities Agency. Authors MJD, CGR, and JLAW have consultancy agreements with Stryker, a commercial party indirectly related to this article. Previous unrestricted research grants have been received from Stryker, Zimmer, and Wright Medical Technologies Inc. by the institution with which MJD, CGR, and EKL are associated.

## Results

### Subjects

518 primary TKA with RSA markers inserted were available from the Halifax RSA Database. After cases were removed under the exclusion criteria or due to missed follow-up visits (Figure 1 and Table 1, see Supplementary data) 360 primary TKA in 333 individuals were analyzed; 222 knees had cemented tibial baseplates and 138 were uncemented. For bone and implant markers, conditions numbers were 36 (19) and 37 (20) (mean [SD]) respectively while mean error of rigid body fitting was 0.13 mm (0.07) mm and 0.10 mm (0.07) respectively. Double exam precision was calculated for all available cases (Table 4, Supplementary data). Surgeries were performed by 7 surgeons and 8 implant designs were used (5 uncemented, Table 6). Simplex P bone cement (Stryker, Mahwah, NJ, USA) was used for all cemented components.

Comparing demographics between the cemented and uncemented groups (Table 6), the uncemented group had a lower mean BMI (p < 0.001, t-test) and a higher proportion of male subjects (p < 0.001, Fisher’s exact test).

### 1-year migration

Tibial component migration measured as MTPM at 1 year was lower for the cemented group compared with the uncemented group (p < 0.001 unadjusted and adjusted for age, sex, BMI; Figure 2, Table 7).

**Figure 3.**
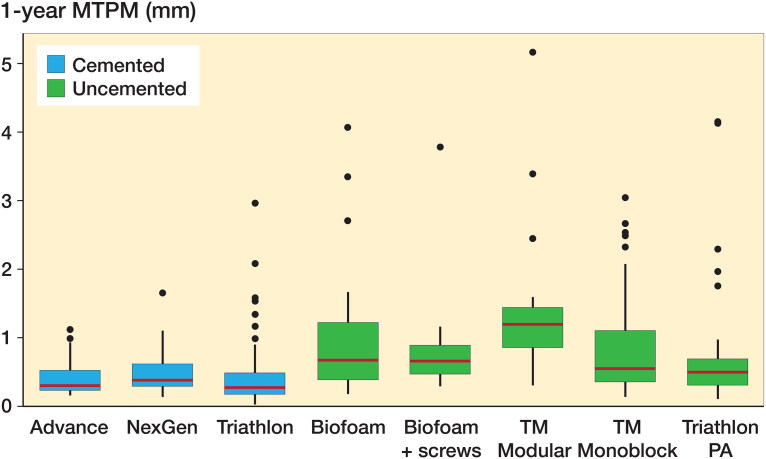
1-year MTPM migration for cemented and uncemented tibial components by implant design. Boxes enclose 25th–75th percentiles with internal horizontal line at the median, whiskers extend a further 1.5 times the inter-quartile range and points beyond this range are plotted individually.

Within the cemented group, the NexGen implant group had greater 1-year migration (p = 0.03 unadjusted and adjusted for age, sex, BMI; Figure 3). For the uncemented implants, the TM Modular implant demonstrated higher 1-year migration (unadjusted: p-value < 0.001; adjusted for age, sex, BMI: p-value = 0.006; Figure 3).

### Change between 1- and 2-year migration

**Figure 4.**
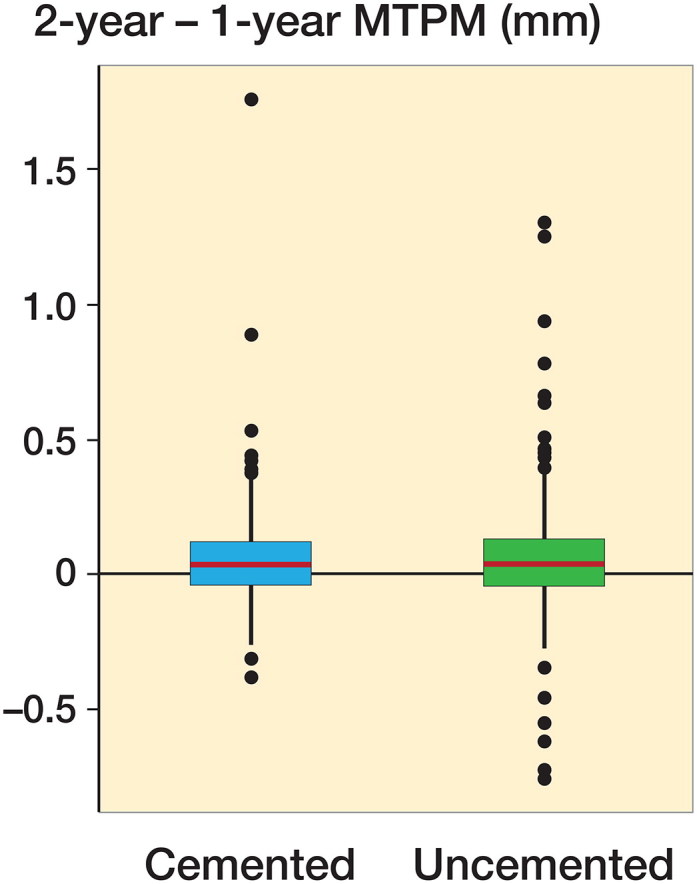
Change in MTPM migration from 1 to 2 years by fixation (cemented, n = 222; uncemented, n = 138). Boxes enclose 25th–75th percentiles with internal horizontal line at the median, whiskers extend a further 1.5 times the inter-quartile range and points beyond this range are plotted individually.

The change in MTPM migration between 1 and 2 years was not statistically significantly different between the cemented and uncemented groups (unadjusted: p-value = 0.7; adjusted for age, sex, BMI: p-value = 0.6; Figure 4, Table 7). The proportion of implants with continuous migration between 1 and 2 years of more than 0.2 mm was similar between groups, with 29/221 (13%) in the cemented group and 21/138 (15%) in the uncemented group.

**Figure 5.**
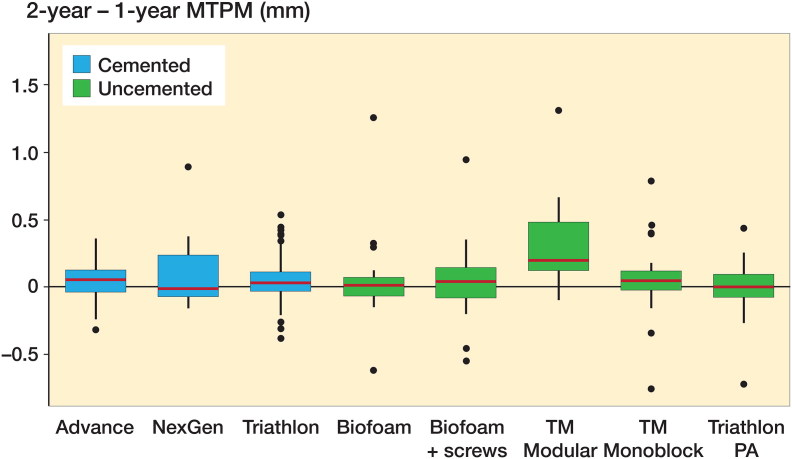
Change in MTPM migration from 1 to 2 years for cemented and uncemented tibial components by implant design. Boxes enclose 25th–75th percentiles with internal horizontal line at the median, whiskers extend a further 1.5 times the inter-quartile range and points beyond this range are plotted individually.

When comparing the change in migration of individual implant designs in the group between 1 and 2 years, the NexGen group had a higher change in migration (p = 0.03 unadjusted and adjusted for age, sex, BMI; Table 7, Figure 5). The NexGen group contained 1 outlier, defined as having a change in MTPM of more than 2 SD from the mean. Removing this subject did not alter the overall conclusion of the analysis (cemented and uncemented implants had similar change in migration between 1 and 2 years), but within the cemented group the NexGen group no longer had statistically significantly different migration for both the 1-year migration value and the change in migration from 1 to 2 years.

Of the 5 implant designs in the uncemented group, the TM Modular group had a greater change in migration from 1 to 2 years compared with the other implant designs (p < 0.001 unadjusted and adjusted for age, sex, BMI; Table 7, Figure 5). Additionally, of the continuous migrators, 8 were TM Modular implants, representing half of this implant group. As the results for the TM Modular group suggested poorer long-term outcomes, there was concern that this group was influencing the overall results for the uncemented group. To investigate this, the TM Modular group (n = 16) was excluded and the data reanalyzed. Using a modified uncemented group did not alter the conclusions: MTPM migration at 1 year remained significantly higher for the modified uncemented group (median = 0.57 mm, range 0.11–4.17 mm, n = 122) compared with the unchanged cemented group (p < 0.001 unadjusted and adjusted for age, sex, BMI) and the change in MTPM migration between 1 and 2 years for the modified uncemented group (mean [SD] 0.03 mm [0.24]) was similar to that for the cemented group (p = 0.4 unadjusted and adjusted).

## Discussion

The application of equivalent thresholds of acceptable RSA migration at 1 year for cemented and uncemented TKA appears to be suboptimal, as higher initial migration seen in the first postoperative year for uncemented components was not associated with greater migration between 1 and 2 years, which is an established criterion for predicting longer-term fixation (Ryd et al., 1995).

Pooling RSA data of both cemented and uncemented tibial components has been employed in 2 important previous studies using early RSA data to predict long-term implant outcomes. In the first study, Ryd et al. (1995) found that MTPM migration between 1 and 2 years postoperatively of greater than 0.2 mm was predictive of later loosening with 85% predictive power. 158 cases were included in that analysis, composed of 103 cemented components and 55 uncemented components. In the second study, Pijls et al. (2012b) in a meta-analysis concluded that mean MTPM migration at 1 year of greater than 0.5 mm put an implant design “at risk” and greater than 1.6 mm was “unacceptable” based on the predicted revision rate at 5 years. In that meta-analysis, 847 subjects were included from 50 RSA studies that were matched to survival studies of the same implant designs (20,599 subjects in 56 studies). Of the 28 implant designs included, 18 had cemented fixation and 10 were uncemented.

The differences found in our study at 1 year are statistically significant and clinically relevant because the differences in means place the cemented group, as well as each cemented implant design, in the “stable” category and the uncemented group, and all individual uncemented implant designs, in the “at risk” category according to Pijls et al. (2012b). However, our findings of equivalent change in migration between 1 and 2 years indicate no greater risk for uncemented implants despite greater uncertainty at 1 year based on the current threshold. The value of increased certainty by 1 year is that in a model of phased innovation (Malchau 2000, Nelissen et al. 2011, Pijls 2014), an early time point for safety thresholds substantially reduces the follow-up time required, providing more timely assessment of implant designs and limiting exposure.

Higher 1-year migration for uncemented implants may be due to a “settling” period prior to bone ingrowth (Onsten et al. 1998, Molt and Toksvig-Larsen 2014, Henricson and Nilsson 2016). Once osseointegration is achieved, the potential for long-term fixation is good for uncemented tibial components while cemented components are susceptible to cement-related complications such as cement delamination (Dalury 2016). Previous RSA studies comparing cemented and uncemented implants have reported higher early migration for the uncemented components while achieving good long-term performance with contemporary uncemented fixation, including hydroxyapatite coatings and trabecular metal monoblock components (Hilding et al. 1995, Nilsson et al. 1999, Regner et al. 2000, Toksvig-Larsen et al. 2000, Carlsson et al. 2005, Nilsson et al. 2006, Pijls et al. 2012a, Wilson et al. 2012, van Hamersveld et al. 2017, Pijls et al. 2018). Review papers of cemented versus uncemented fixation have been inconclusive, citing a lack of long-term follow-up studies, but do conclude that there are promising results, especially with hydroxyapatite coatings and trabecular metal in short-term and RSA studies (Nakama et al. 2012, Brown et al. 2013, Mont et al. 2014). The 1-year MTPM migrations in the current study are similar to those found in a meta-analysis of tibial component migration for cemented (mean MTPM of 0.44 mm) and uncemented components (mean MTPM of 1.09 mm) (Pijls et al. 2018). The authors additionally proposed using the previously published 1-year MTPM migration thresholds at 6 months instead due to minimal migration between 6 months and 1 year (Pijls et al. 2018), but do not suggest differing thresholds for cemented and uncemented fixation as we and other authors have previously suggested (Henricson and Nilsson 2016).

We found statistically significant differences in 1- to 2-year migration between different types of uncemented fixation, suggesting that not all uncemented fixation is equivalent. The TM Modular implant group had the least favorable migration results with significantly greater migration at both 1 year and between 1 and 2 years, indicating that this implant design is at greater risk of poor long-term survivorship. While both the TM Modular and TM Monoblock tibial components rely on bone in-growth into a porous trabecular metal structure, the benefits of the lower modulus monoblock component may be compromised with the addition of a stiff baseplate in the modular component to allow polyethylene inserts to be locked in place. Previous studies on the TM Modular component have reported 4 failures due to aseptic loosening in 167 cases (2%, all within the first postoperative year) (Zandee van Rilland et al. 2015); 7 revised or radiographically loose components in a series of 51 subjects (Behery et al. 2017); 1 revision for subsidence out of 50 cases (Fricka et al. 2015); and statistically significantly higher overall migration compared with the TM Monoblock component, but no difference between groups in change in migration from 1 to 2 years in 53 subjects (Andersen et al. 2016). It has not been possible to date to identify the TM Modular component in isolation in any national knee registry reports, so the survivorship of this implant in general use remains to been seen. Notably, a similar uncemented implant design by the same manufacturer employing trabecular metal and a modular tibial tray was recalled in 2015 due to an increase in complaints of loosening and radiolucent lines (FDA 2015).

Additionally of note, the uncemented group with screw fixation performed equivalently to the same implant without screw fixation, although the intention of screw fixation is to provide immediate stability. Lack of immediate stability with screw fixation has been seen in previous RSA studies (Nilsson et al. 2006, Stilling et al. 2011).

The differences in magnitudes between the uncemented subgroups may offer a preview of refined thresholds for 1-year screening of uncemented implants: the median 1-year migration of the TM Modular group was 1.2 mm compared with 0.5–0.7 mm for the other four uncemented groups. Matching of RSA and survivorship studies will be required to perform the robust analysis of Pijls et al. (2012b) to determine if a separate early (6-month or 1-year) threshold for uncemented components is valid. Alternatively, using a later reference exam (such as 6 weeks or 3 months) may permit determination of an early threshold that is valid for both cemented and uncemented fixation, but may be more difficult to find evidence for from the available literature.

A limitation of our study is that subjects were not randomly assigned to the cemented and uncemented groups. However, this study represents analysis of 1 of the largest datasets of TKA postoperative RSA data to date. The results therefore provide important insight mechanisms into early migration depending on implant fixation. The demographic data show statistical differences between groups, although the clinical relevance of a BMI difference of 2 (with both groups > 30) is likely negligible. The proportion of females in the cemented group (68%) versus the uncemented group (49%) was unexpected and may reflect an unconscious bias by operating surgeons in not using uncemented implants in women due to bone quality concerns. These demographic variables were accounted for in the statistical models, so the differences between fixation methods cannot be attributed to mismatched demographic factors between the cemented and uncemented groups. Although demographic factors are eclipsed by the differences due to implant fixation method in the overall group, it is likely that demographic factors do influence implant migration and may account for some of the variability in early migration, especially within the uncemented group.

We excluded revised implants in this study to allow a comparison of the 2 methods for thresholds of allowable motion. Of the 14 total revisions, only 3 were performed for reasons related to mechanical loosening (1 peri-prosthetic fracture and 2 for aseptic loosening) and all 3 revisions were performed within the first 2 postoperative years so these cases would have been excluded from the analysis by default as the change in migration from 1 to 2 years could not be evaluated. Excluding the remaining cases ensured that no additional cases of mechanical loosening were included as our data capture only the most responsible reason for revision in what may be a multifactorial process.

In summary, our study finds that the pattern of migration between 1 and 2 years was similar between cemented and uncemented groups and therefore supports both the use of uncemented fixation and the previous findings that this metric is appropriate to evaluate all tibial component fixations (Ryd et al. 1995). However, the magnitudes of migration at 1 year are higher for the uncemented group suggesting that thresholds at 1 year may not apply equally to cemented and uncemented implants for predicting revision rates as suggested by Pijls et al. (2012b). A further refinement of the 1-year threshold may be appropriate for uncemented implants to enable more conclusive evaluations of uncemented implant designs.

## Supplementary Material

Supplemental Material
