# Supplementary material for: Equivalent 2-year stabilization of uncemented tibial component migration despite higher early migration compared with cemented fixation: an RSA study on 360 total knee arthroplasties
Source: Acta Orthop. 2019 Jan 23;90(2):172–8. doi: 10.1080/17453674.2018.1562633 (PMC6461081; doi:10.1080/17453674.2018.1562633)
Supplement: Supplemental Material [file IORT_A_1562633_SM5799.pdf]

Supplementary data

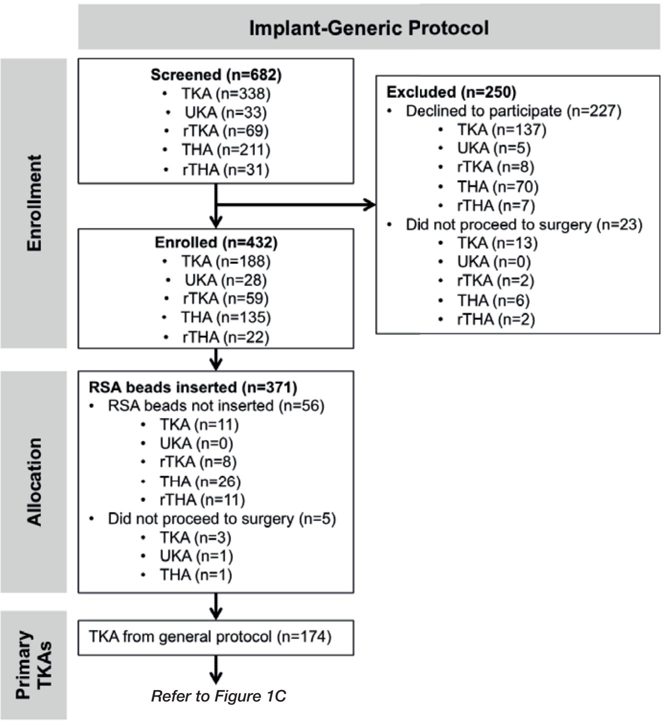

Figure 1A. Part 1/3 of Consort Diagram for all TKA subjects: subjects from implant-generic protocol. UKA = unicondylar knee arthroplasty, rTKA = revision total knee arthroplasty, THA = total hip arthroplasty, rTHA = revision total hip arthroplasty.

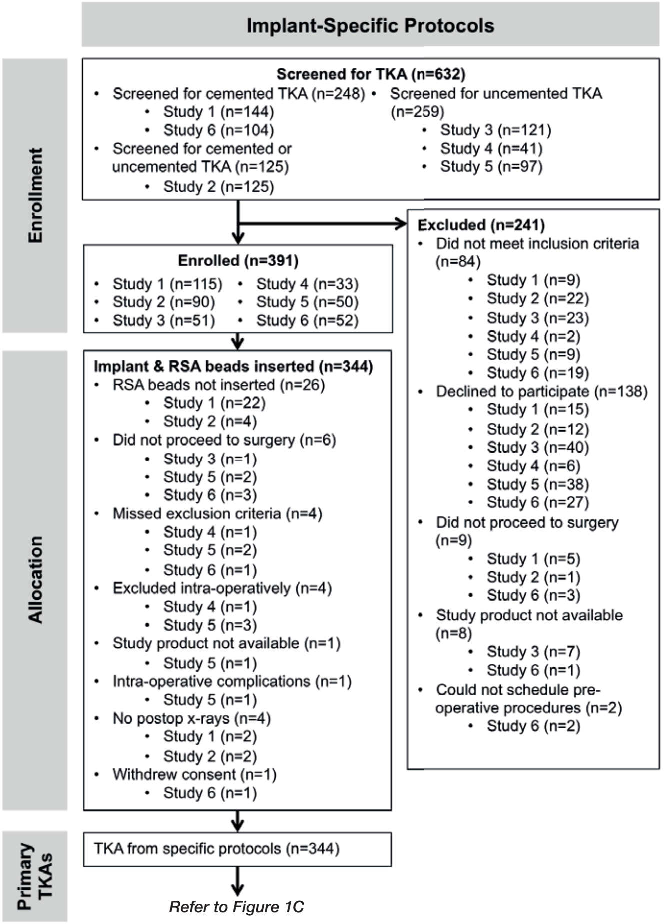

Figure 1B. Part 2/3 of Consort Diagram for all TKA subjects: subjects from implant-specific protocols. Refer to Table 2 for study details.

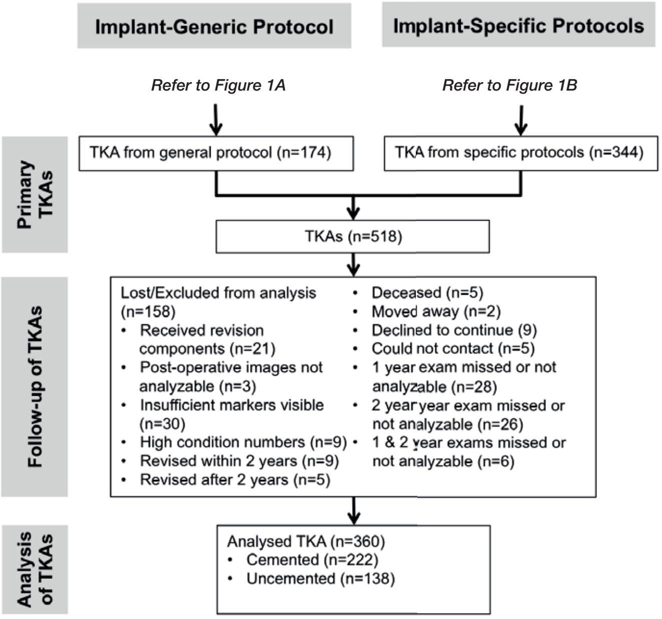

Figure 1C. Part 3/3 of Consort Diagram for all TKA subjects

Table 1. Revised tibial components by fixation and time frame

	Cemented	Uncemented
**Revision within 2 years of surgery (n = 9)**		
Revision related to mechanical loosening (n = 1 + 2)		
	aseptic loosening, 1	aseptic loosening, 1
		peri-prosthetic fracture, 1
Revision not related to mechanical loosening (n = 4 + 2)		
	infection, 3	infection, 2
	instability, 1	
**Revision more than 2 years from surgery (n = 5)**		
Revision related to mechanical loosening (n = 0)		
Revision not related to mechanical loosening [a] (n = 3 + 2)		
	instability, 2	pain, 1
	pain, 1	infection, 1

[a] Mean time to revision = 3.3 (2.2–6.1) years
Note: only a single "most responsible" reason for revision is documented.

Table 2. Study details, ethics and clinical trial registration numbers

Implant-generic protocol
"Development of a Clinical Diagnostic System for Assessing Orthopaedic Implant Stability"
• Local ethics approval numbers: CDHA-RS/2010-388 [a], 1020265 [b]
• ClinicalTrials.gov identifier: N/A
• Sources of funding: Atlantic Canada Opportunities Agency Atlantic Innovation Fund

Implant-specific protocols
Study 1: "A Prospective Randomized Trial using Roentgen Stereophotogrammetric Analysis of the Advance Medial Pivot Knee"
• Local ethics approval numbers: CDHA-RS/2001-213 [a]
• ClinicalTrials.gov identifier: NCT00405470
• Source of funding: Wright Medical Technologies

Study 2: "A Prospective Randomized Controlled Trial using Roentgen Stereophotogrammetric Analysis (RSA) of a Trabecular Metal Mesh Tibial Monoblock Knee Arthroplasty Component"
• Local ethics approval numbers: CDHA-RS/2002-096 [a]
• ClinicalTrials.gov identifier: NCT00405379
• Source of funding: Zimmer

Study 3: "Prospective Clinical Study using Roentgen Stereophotogrammetric Analysis (RSA) and DEXA to Evaluate Fixation of Periapatite coated Triathlon Total Knee Arthroplasty Components"
• Local ethics approval numbers: CDHA-RS/2009-039 [a], 1020606 [b], Refereneence Number 293 (Protocol TRI-DC-06) [c]
• ClinicalTrials.gov identifier: NCT01180582
• Source of funding: Stryker

Study 4: "A Prospective RCT using Roentgen Stereophotogrammetric Analysis (RSA) to Evaluate Fixation of the Biofoam Advance Total Knee Arthroplasty Components with and without Screw Augmentation"
• Local ethics approval numbers: CDHA-RS/2007-250 [a], 1020650 [b]
• ClinicalTrials.gov identifier: NCT00657956
• Source of funding: Wright Medical Technologies

Study 5: "Randomized Control Trial Using Radiostereometric Analysis (RSA) to Compare the Fixation of the Trabecular Metal Monoblock and the Trabecular Metal Modular Total Knee Arthoplasties"
• Local ethics approval numbers: CDHA-RS/2011-010 [a], 1000199 [b]
• ClinicalTrials.gov identifier: NCT01180595
• Sources of funding: Zimmer, ACOA/AIF

Study 6: "Randomized Control Trial using RSA to Compare the OtisMed Customfit Total Knee Replacement Procedure with Computer Assisted Surgery"
• Local ethics approval numbers: CDHA-RS/2011-296 [a], 1005885 [b]
• ClinicalTrials.gov identifier: NCT01262430
Sources of funding: Stryker

Study 7: "A Ten-year Evaluation of Implant Fixation in Four Total Knee Replacement Designs using Radiostereometric Analysis"
• Local ethics approval numbers: CDHA-RS/2015-229 [a], 1018407 [b]
• ClinicalTrials.gov identifier: N/A
• Sources of funding: NSHARF
Note: this study is a follow-up study of subjects enrolled in Study 1 & Study 2 above and therefore does not represent any additional subjects

[a] Capital District Health Authority Research Ethics Board (Halifax, Nova Scotia, Canada)
[b] Nova Scotia Health Authority Research Ethics Board (Halifax, Nova Scotia, Canada) (note: name change from Capital District Health Authority Research Ethics Board (effective April 1, 2015) but the same institution)
[c] St John of God Health Care Ethics Committee (Subiaco, Perth, Western Australia, Australia)

Table 3. RSA equipment details

Calibration boxes
1. Biplanar calibration box (Tilly Medical Products AB, Lund, Sweden), 90° between beams (Halifax, 2002–2003)
2. Uniplanar calibration box (Halifax Carbon Box, MEDIS medical imaging systems BV, Leiden, The Netherlands), 1.6 m from calibration box to X-ray heads, beams angled 20° from the vertical (Halifax, 2003–March 2008 and September 2009–July 2010)
3. Uniplanar calibration box (HBI Box003 Halifax, Halifax Biomedical Inc., Mabou, Nova Scotia, Canada), beams angled 30° from vertical (Halifax, March 2008–August 2009)
4. Uniplanar calibration box (Perth Carbon Box, MEDIS medical imaging systems BV, Leiden, The Netherlands), and X-ray beams angled 20º from the vertical. (Perth, 2009–2010)
5. Uniplanar calibration box (HBI Box007 Halifax, Halifax Biomedical Inc., Mabou, Nova Scotia, Canada), beams angled 30° from vertical (Halifax, 2010 onwards)

X-ray heads
1. 1 fixed X-ray head (Model Ultranet-SA, GE Medical Systems, Monza, Italy) and 1 portable X-ray head (Model 46-194759G1, General Electric Company, Milwaukee, WI, USA) (Halifax, 2002–2008)
2. 2 ceiling mounted X-ray tubes (Rad92, Varian Medical Systems, Salt Lake City, UT, USA) (Halifax, 2008 onwards)
3. 1 ceiling mounted tube (Toshiba DST-100A, Japan) and 1 portable X-ray machine (GE Medical Systems AMX4 XFMR, Milwaukee, WI, USA) (Perth, 2009–2010)

Cassettes and detectors
1. AFGA-Gevaert NV CRMD4.0 cassettes (35 x 43 cm) (Mortsel, Belgium) scanned with AGFA-Gevaert NV CR85-X digitizer (Mortsel, Belgium) producing images with a spatial resolution of 6 pixels/mm and greyscale resolution of 12 bits/pixel (Halifax, 2002–2008 and September 2009–July 2010)
2. IDC X1590 DR SYSTEM X4C digital detectors (Imaging Dynamics Company Ltd., Calgary, Alberta, Canada), 43 x 43 cm, pixel size 108 microns^2 (Halifax, March 2008–August 2009)
3. Kodak GP Storage Phosphor System 35x43 cm cassettes (Carestream Health, Inc., Rochester, NY, USA) with Kodak Directview CR850 System digitizer (Carestream Health, Inc., Rochester, NY, USA) producing images with a spatial resolution of 5.8 pixels/mm and a 12-bit grayscale resolution (Perth, 2009–2010)
4. CXDI-55C digital detectors (Canon Inc., Tokyo, Japan), 35 x 43 cm (2,208 x 2,688 pixels), pixel size 160 microns^2, greyscale resolution of 12 bits/pixel (Halifax, 2010 onwards)

RSA beads
1. Tantalum RSA marker beads (0.8 mm in diameter; Wennbergs Finmek AB, Gunnilse, Sweden) (2002–2004)
2. Tantalum RSA marker beads (1.0 mm in diameter; Halifax Biomedical Inc., Mabou, NS, Canada) (2004 onwards)

RSA Software
1. RSA-CMS, Version 4.3, MEDIS medical imaging systems BV, Leiden, The Netherlands (2002–2004)
2. Model-based RSA (Version 3.21, Medis specials b.v., Leiden, The Netherlands)
3. Model-based RSA (Version 3.32, Version 3.4, RSAcore, Leiden, The Netherlands) (2004 onwards)

RSA analysis support
1. Halifax Biomedical Inc. (Mabou, NS, Canada)

Table 4. RSA Precision calculated from 267 double exams

	mean	SD	Precision (1.96*SD) [a]
Translations (mm)			
x	0.00	0.04	0.08
y	0.00	0.04	0.07
z	0.01	0.08	0.15
Rotations (degrees)			
Rx	0.00	0.16	0.31
Ry	0.00	0.09	0.17
Rz	0.00	0.07	0.14
MTPM (mm)	0.13	0.07	0.14

Anatomical directions for reported translations and rotations as follows: x translation: medial (+) / lateral (–); y translation: superior (+) / inferior (–); z translation: anterior (+) / posterior (–); x rotation: anterior tilt (+) / posterior tilt (–); y rotation: internal (+) / external (–); z rotation: adduction (+) / abduction (–)
[a] ISO 16087: 2013 Implants for surgery -- Roentgen stereophotogrammetric analysis for the assessment of migration of orthopedic implants.

Table 5. Results for the effect of fixation and implant on 1-year MTPM migration (mm) and change in migration from 1 to 2 years (MTPM) for all tibial components and for cemented and uncemented implants separately, adjusted for sex, age and BMI where noted

	Estimate	SE [a]	95% CI [b]	p-value
**1-year migration (log_{10}(MTPM) as outcome variable)**				
All tibial components (cemented and uncemented)				
Unadjusted model				
Fixation	0.33	0.04	0.26–0.40	< 0.001
Adjusted model				
Fixation	0.34	0.04	0.27–0.41	< 0.001
Sex	0.04	0.04	–0.03–0.11	0.3
Age	0.00	0.00	0.00–0.01	0.1
BMI	0.00	0.00	0.00–0.01	0.3
Uncemented tibial components				
Unadjusted model				
Biofoam	0.12	0.10	–0.07–0.30	0.2
Biofoam + Screws	0.06	0.10	–0.13–0.25	0.6
TM Modular	0.36	0.10	0.15–0.56	<0.001
TM Monoblock	0.03	0.08	–0.12–0.19	0.7
Adjusted model				
Biofoam	0.05	0.10	–0.14–0.24	0.6
Biofoam + Screws	-0.01	0.10	–0.20–0.18	0.9
TM Modular	0.30	0.11	0.09–0.51	0.01
TM Monoblock	-0.01	0.08	–0.17–0.14	0.9
Sex	0.07	0.06	–0.05–0.19	0.3
Age	0.01	0.00	0.00–0.02	0.04
BMI	0.01	0.01	0.00–0.02	0.08
Cemented tibial components				
Unadjusted model				
Advance	0.07	0.05	-0.02–0.16	0.2
NexGen	0.13	0.06	0.01–0.25	0.03
Adjusted model				
Advance	0.07	0.05	-0.03–0.16	0.2
NexGen	0.13	0.06	0.01–0.25	0.03
Sex	0.00	0.05	-0.09–0.09	1.00
Age	0.00	0.00	0.00–0.01	0.7
BMI	0.00	0.00	-0.01–0.01	0.9

	Estimate	SE [a]	95% CI [b]	p-value
**Change in migration 1 to 2 years (MTPM as outcome variable)**				
All tibial components (cemented and uncemented)				
Unadjusted model				
Fixation	0.01	0.02	–0.04–0.06	0.7
Adjusted model				
Fixation	0.01	0.03	–0.04–0.06	0.6
Sex	–0.01	0.03	–0.06–0.04	0.6
Age	0.00	0.00	0.00–0.00	0.5
BMI	0.00	0.00	0.00–0.01	0.1
Uncemented tibial components				
Unadjusted model				
Biofoam	0.06	0.07	–0.08–0.21	0.4
Biofoam + Screws	0.05	0.07	–0.09–0.20	0.5
TM Modular	0.33	0.08	0.17–0.49	< 0.001
TM Monoblock	0.06	0.06	–0.06–0.18	0.3
Adjusted model				
Biofoam	0.07	0.08	–0.08–0.22	0.4
Biofoam + Screws	0.05	0.08	–0.10–0.20	0.5
Implant: TM Modular	0.35	0.09	0.19–0.52	<0.001
Implant: TM Monoblock	0.08	0.06	–0.05–0.20	0.2
Sex	–0.03	0.05	–0.13–0.06	0.5
Age	0.00	0.00	–0.01–0.01	0.7
BMI	0.00	0.00	–0.01–0.01	0.7
Cemented tibial components				
Unadjusted model				
Advance	–0.01	0.03	–0.07–0.05	0.8
NexGen	0.08	0.04	0.01–0.16	0.03
Adjusted model				
Advance	0.00	0.03	–0.06–0.06	1.0
Prosthesis: NexGen	0.08	0.04	0.01–0.16	0.03
Sex	–0.01	0.03	–0.06–0.05	0.82
Age	0.00	0.00	0.00–0.00	0.39
BMI	0.00	0.00	0.00–0.01	0.14

Reference levels for factor variables: fixation: cemented; sex: male, uncemented implant: Triathlon PA (lowest median 1-year MTPM migration); cemented implant: Triathlon (lowest median 1 year MTPM migration)
[a] SE = Standard error
[b] CI = 95% confidence interval, two-tailed.
